# Two new species of *Centrodora* (Hymenoptera, Aphelinidae) from China, with a key to Chinese species

**DOI:** 10.3897/zookeys.687.13164

**Published:** 2017-08-01

**Authors:** Ye Chen, Cheng-De Li

**Affiliations:** 1 School of Forestry, Northeast Forestry University, Harbin, 150040, China

**Keywords:** Aphelininae, Aphytini, Chalcidoidea, taxonomy

## Abstract

Two new species of *Centrodora* Förster, *C. crassiscapa* **sp. n.** and *C. pellucida* **sp. n.**, are described from China. A key to species from China based on females is given.

## Introduction

*Centrodora* Förster, 1878 currently comprises 61 species (Noyes 2017). A majority of the species in this genus are oophagous, developing in the eggs of economically important Hemiptera and Orthoptera, whereas other species are known to attack nymphs of Hemiptera, and pupae of Diptera, Hymenoptera, and Coleoptera (Hayat 1974, 2010; Polaszek 1991). Several regional revisionary studies of the genus include Mercet (1918, 1930) and Nikol’skaya and Yasnosh (1966) on the European region, Hayat (1998, 2010) on the Indian region.

The Chinese fauna of *Centrodora* is poorly known with only *C. lineascapa* Hayat known from the Fujian Province (Huang 1994), and *C. idioceri* Ferrière (Chou and Chou 1990) and *C. locustarum* (Giraud) (Chiu and Chou 1974) known from Taiwan. In the present paper, two new species are added to the Chinese fauna. A key to all five species from China based on females is given.

## Materials and methods

All specimens in the present study were collected using yellow pan traps or Malaise traps, then dissected and mounted in Canada Balsam on slides following the method described by Noyes (1982). Photographs were taken with a digital CCD camera attached to an Olympus BX51 compound microscope and final modifications to the images were made using Adobe Photoshop. Most measurements were made from slide-mounted specimens using a reticle micrometre except the total body length (excluding the ovipositor), which was measured from alcohol-preserved specimens before being dissected. All measurements are given in micrometres (μm) except body length, which is measured in millimetres (mm). Scale bars are 100 μm. All the specimens listed below are deposited in Northeast Forestry University, Harbin, China. Terminology follows Hymenoptera Anatomy Consortium (2017).

**The following abbreviations are used in the text**

**F1−3** funicle segments 1−3;

**TI, TII etc.** tergites 1, 2, etc. of gaster;

**MT** Malaise trap.

**The following acronyms are used for the depositories**

**NEFU** Northeast Forestry University, Harbin, China.

## Results

### Key to Chinese species (female) of *Centrodora* Förster

**Table:** 

1	Clava with apex rounded, not curved ventrally; antenna with F3 distinctly longer than wide	***C. idioceri* Ferrière**
–	Clava with apex pointed and curved ventrally (Figs 2, 13)	**2**
2	Ovipositor strongly exerted and with the exerted part more than 0.3× as long as ovipositor (Figs 8, 19)	**3**
–	Ovipositor very slightly exerted	**4**
3	Scape 3.26× as long as wide, remarkably wider than any other antennal segments (Fig. 2); median area of the mesoscutum and mesoscutellum dark brown; fore wing (Fig. 5) with basal one fourth hyaline, area beneath marginal vein brown, remaining part faintly infuscate	***C. crassiscapa* sp. n.**
–	Scape (Fig. 13) 3.58−4.13× as long as wide; median area of the mesoscutum and scutellum yellowish-brown; fore wing mostly infuscate, with a transparent cross band on the median area of disc as in Fig. 16	***C. pellucida* sp. n.**
4	Gaster completely blackish, hind leg with coxa and femur black	***C. locustarum* (Giraud)**
–	Gaster with yellow and brown tergites, hind leg completely pale	***C. lineascapa* Hayat**

#### Centrodora crassiscapa

Taxon classificationAnimaliaHymenopteraAphelinidae

Li & Chen sp. n.

http://zoobank.org/7A12C8EE-F827-444F-915F-D9FDA725D5F6

Figs 1–8 , 9–11

##### Type material.

**Holotype**: female [on slide, NEFU], CHINA, Heilongjiang Province, Shangzhi City, Laoyeling (45°24.71'N, 127°40.41'E), 8–18.VII.2013, Cheng-de Li, Ye Chen, Chao Zhang, MT. **Paratypes**: 1 male [on slide, NEFU], same data as holotype. 1 male [on slide, NEFU], CHINA, Heilongjiang Province, Shangzhi City, Laoyeling, 7–16.VIII.2013, Cheng-de Li, MT.

##### Diagnosis.

*Centrodora crassiscapa* sp. n. is easily distinguished by following combination of characters: enormously thick scape, largely dark brown mesosoma, completely dark brown metasoma, long and strongly exserted ovipositor.

##### Description.

***Female***. Holotype. Length about 1.2 mm. Head dark brown. Eyes and ocelli dark brown. Antenna mostly brown, except pedicel and F1 paler. Mandible dark brown. Mesosoma largely dark brown, except metanotum and propodeum yellow. Median area of the mesoscutum and mesoscutellum with a distinctly pale mid-longitudinal groove. Fore wing with basal one fourth hyaline, area posterior to marginal vein brown, remaining wing disc faintly infuscate; hind wing hyaline. Legs largely yellow, except fore leg with distal half of femur and tibia brownish-yellow; mesofemur and hind leg with coxa and femur brown. Metasoma dark brown to black.

**Figures 1–8.**
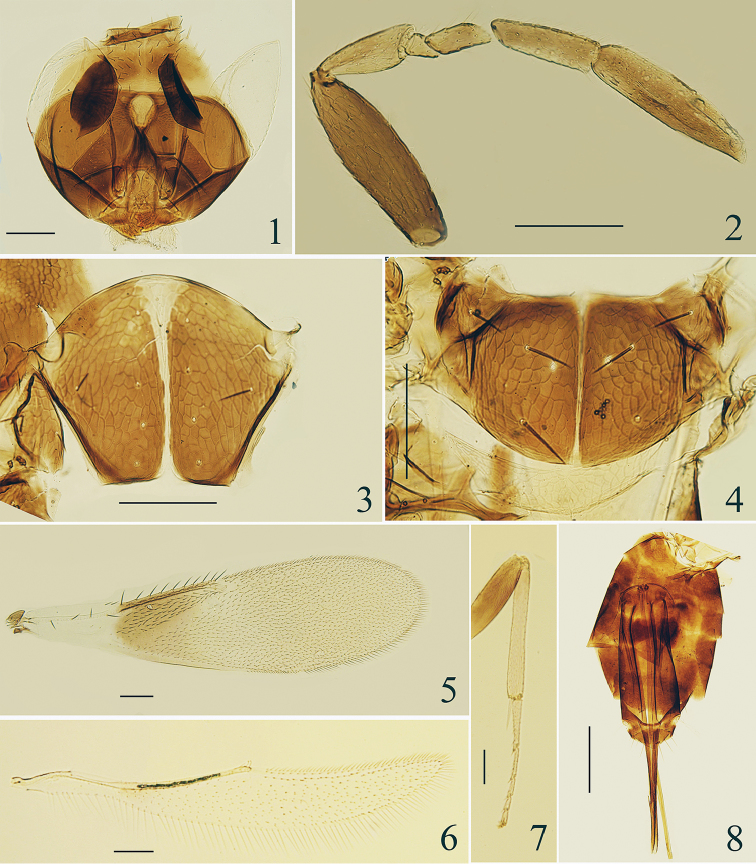
*Centrodora crassiscapa* sp. n., holotype female: **1** head, frontal view **2** antenna **3** mesoscutum **4** mesoscutellum and metanotum **5** fore wing **6** hind wing **7** mesotibia and tarsus **8** metasoma.

*Head* (Fig. 1), in frontal view, 0.95× as high as wide, and finely reticulate, with the sculpture on malar space lineolate reticulated. Frontovertex 0.43× head width and with numerous brown setae. Ocellar triangle with apical angle obtuse. Mandible with two acute teeth and a blunt dorsal tooth. Antennae (Fig. 2) with scape 3.26× as long as wide, remarkably wider than any other antennal segments and slightly longer than clava; pedicel 2.14× as long as wide, and 0.83× as long as F3; F1 subtriangular, with ventral margin 1.40× as long as wide; F2 2.0× as long as wide; F3 with dorsal margin 3.21× as long as wide, and 0.6× as long as clava; clava 3.95× as long as wide, pointed and distinctly curved ventrally at apex. Measurements, length (width): scape, 163 (50); pedicel, 75 (35); F1, 28 (20); F2, 40 (20); F3, 90 (28); clava 150 (38).

*Mesosoma*. Pronotum imbricate, mesoscutum and mesoscutellum mainly polygonal reticulate. Mesoscutum with median area (Fig. 3) 0.83× as long as wide, 1.4× as long as mesoscutellum, and with 10 setae; each lateral area of the mesoscutum and axilla with 2 and 1 setae respectively; mesoscutellum (Fig. 4) 0.58× as long as wide, with 2 pairs of setae. Distance between anterior pair of scutellar setae 0.9× that between posterior pair. Placoid sensilla much closer to anterior pair of setae than to posterior pair. Fore wing 3.57× as long as wide, with marginal setae 0.08× wing width. Costal cell slightly longer than marginal vein, with 2 distal setae on dorsal surface; submarginal vein with 4 setae, marginal vein with 8 long setae along anterior margin, postmarginal vein short and about one third of length of stigmal vein; basal cell with 2 setae. Linea calva closed by a line of setae posteriorly. Hind wing (Fig. 6) 7.17× as long as wide, marginal setae about 0.53× as long as wing width. Mesotibial spur (Fig. 7) distinctly shorter than (0.64×) corresponding basitarsus, and about as long as the second tarsomere. Measurements, length (width): fore wing 1250 (350); costal cell, 270; marginal vein, 260; postmarginal vein, 13; stigmal vein, 39; marginal setae, 28; hind wing, 1075 (150); marginal setae, 80; mesotibia, 390, mesotibial spur, 90, mesobasitarsus, 140.

*Metasoma* (Fig. 8) about 1.75× as long as mesosoma. Ovipositor 3.25× as long as mesotibia and strongly exerted, with the exerted part 0.46× as long as ovipositor. Third valvula 4.18× as long as mesobasitarsus. Length measurements: ovipositor, 1268; third valvula, 585.

***Male***. Length 0.73–0.78 mm. Colour similar to the female, except median area of mesoscutum a little paler.

**Figures 9–11.**
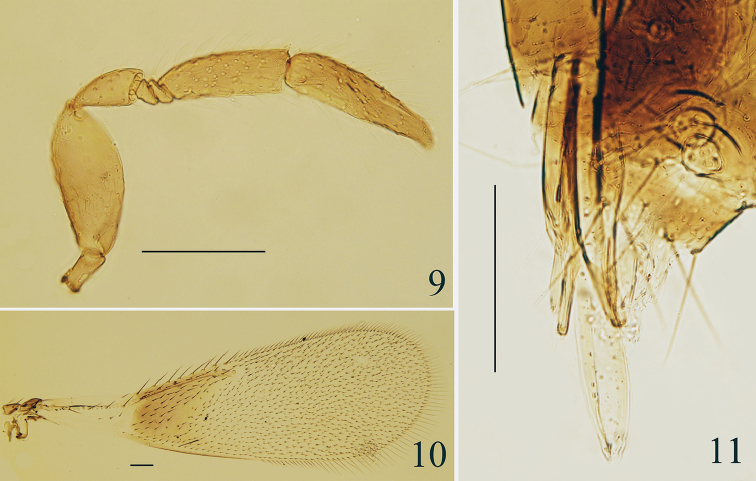
*Centrodora crassiscapa* sp. n., paratype male: **9** antenna **10** fore wing **11** genitalia.

*Head*, in frontal view, 0.9× as high as wide and frontovertex about 0.4× head width. Scape (Fig. 9) flattened and expanded medially, 2.37× as long as wide; pedicel 1.64–1.70× as long as wide; F1 triangular, 1.20× as long as wide, F2 anelliform, F3 long, 2.69–2.85× as long as wide, and 0.71–0.77× as long as clava; clava 3.69–4.08× as long as wide, slightly longer than scape. *Fore wing* (Fig. 10) 3.04× as long as wide, marginal setae 0.17× wing width; hind wing 7.11–7.25× as long as wide, marginal setae 0.63× wing width. *Genitalia* (Fig. 11) 5.30× as long as wide, and about as long as mesotibia.

##### Remarks.

The new species is close to *C. amoena* Förster 1878, with similar colour on the head and metasoma and strongly exserted ovipositor. However, it can be separated from the latter by the thickened scape of the female antennae (normal in *C. amoena*), completely dark brown mesoscutum (*vs* largely yellow), clava 3.95× as long as wide (*vs* 3.5×), male antenna with F3 2.69–2.85× as long as wide (*vs* about 4×, *cf.* Nikol’skaya and Yasnosh 1966, fig. 162).

##### Host.

Unknown.

##### Etymology.

Latin: *crassus* = thick, fat; and the specific name refers to the enormously thick scape of the female antennae.

##### Distribution.

China (Heilongjiang).

#### Centrodora pellucida

Taxon classificationAnimaliaHymenopteraAphelinidae

Li & Chen sp. n.

http://zoobank.org/8FBBCA89-706B-4FA2-8486-55E645648057

Figs 12–19

##### Type material.

**Holotype**: female [on slide, NEFU], CHINA, Heilongjiang Province, Shangzhi City, laoyeling (45°24.71'N, 127°40.41'E), 8–18.VII.2013, Cheng-de Li, Ye Chen, Chao Zhang, MT. **Paratype**: 1 female [on slide, NEFU], CHINA, Shandong Province, Qingdao City, xiaozhu Mountain (35°58.38'N, 120°05.76'E), 18–20.V.2014, Xiang-xiang Jin, Guo-hao Zu, Si-zhu Liu, yellow pan trapping.

##### Diagnosis.

*Centrodora pellucida* sp. n. can be easily distinguished from other species in this genus by the following combination of characters: upper half of head yellowish-brown; gaster dark brown; fore wing mostly infuscate and paler distally, with a wide transparent cross band on the median area of disc as in Fig. 16; F2 subquadrate, and ovipositor strongly exserted.

**Figures 12–19.**
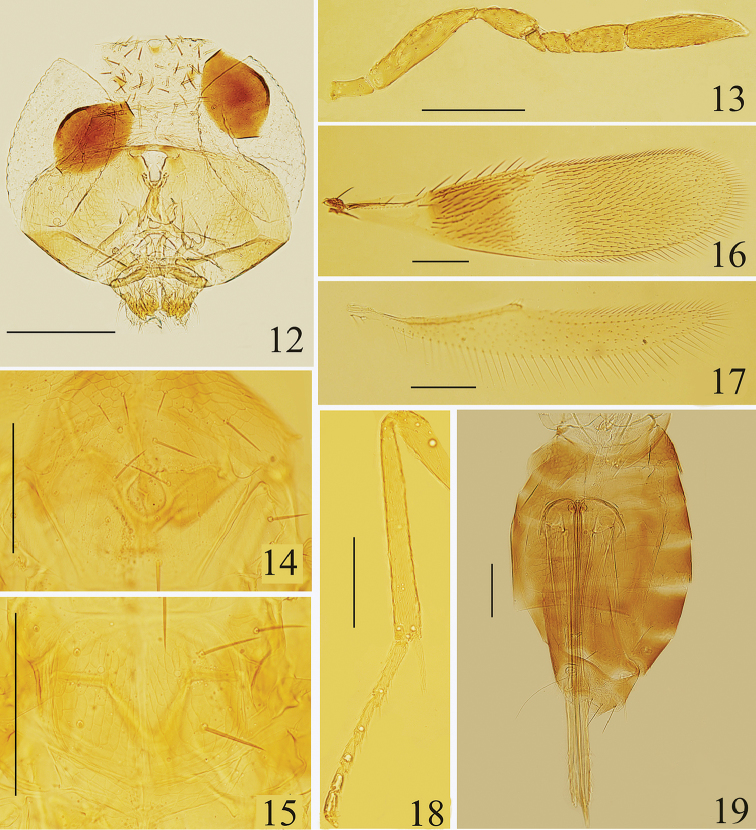
*Centrodora pellucida* sp. n., holotype female: **12** head, frontal view **13** antenna **14** mesoscutum **15** mesoscutellum and metanotum **16** fore wing **17** hind wing **18** mesotibia and tarsus **19** metasoma.

##### Description.

***Female***. Holotype [The colour of body faded during slide mounting, and the following descriptions of colour is based on alcohol-preserved specimens]. Length 0.95 mm. Head with frontovertex, upper face, and occiput above foramen yellowish brown; remainder of head brown. Eyes and ocelli yellowish-brown. Antenna brown. Mandible yellowish-brown, with apex dark brown. Mesosoma largely yellowish-brown, but with mesopleuron brown and with a pale yellow mid-longitudinal groove on median area of mesoscutum, mesoscutellum, metanotum, and propodeum. Fore wing mostly infuscate and paler distally, with a wide transparent cross band on the median area of disc as in Fig. 16. Hind wing faintly infuscate. Leg mostly brownish; mesocoxa paler; meso- and metafemur, both basal one third of meso- and metatibia dark brown. Metasoma mostly dark brown, with petiole and third valvula yellowish.

*Head* (Fig. 12), in frontal view, 0.77× as high as wide; face and malar space finely reticulate. Frontovertex about 0.37× head width, with numerous coarse and brown setae. Ocellar triangle with apical angle about 90°. Mandible with two teeth and a truncation. Antennae (Fig. 13) with scape 4.13× as long as wide, about as long as clava; pedicel 2.0× as long as wide, about as long as F3; F1 triangular, 1.64× as long as wide, with ventral margin very slightly shorter than F2; F2 subquadrate, slightly shorter than half of F3; F3 2.12× as long as wide, 0.44× as long as clava; clava 4.0× as long as wide, pointed and distinctly curved ventrally at apex. Measurements, length (width): scape, 124 (30); pedicel, 50 (25); F1, 23 (14); F2, 25 (23); F3, 53 (25); clava, 120 (30).

*Mesosoma* with fine, elongate reticulations on median area of mesoscutum and mesoscutellum (Figs 14, 15). Median area of mesoscutum 0.93× as long as wide, 1.53× as long as mesoscutellum, and with 10 setae; each lateral area of the mesoscutum and axilla with 2 and 1 setae respectively; mesoscutellum 0.73× as long as wide, with 2 pairs of setae. Distance between anterior scutellar setae subequal to that between posterior pair. Placoid sensilla much closer to anterior pair of setae than to posterior pair. Fore wing (Fig. 16) 4.21× as long as wide, marginal setae 0.22× wing width. Costal cell slightly shorter than marginal vein, with 1 distal seta on dorsal surface; submarginal vein with 4 setae, marginal vein with 6 long setae along anterior margin, postmarginal vein 0.5× as long as stigmal vein; basal cell without seta. Linea calva closed by a line of setae posteriorly. Hind wing (Fig. 17) 6.86× as long as wide, marginal setae 0.67× wing width. Mesotibial spur 0.87× as long as corresponding basitarsus, and 0.83× as long as the second tarsomere. Measurements, length (width): fore wing, 738 (175); costal cell, 160; marginal vein, 170; postmarginal vein, 10; stigmal vein, 20; marginal setae, 38.5; hind wing, 600 (87.5); marginal setae, 59; mesotibia, 250; mesotibial spur, 65; mesobasitarsus, 75.

*Metasoma* (Fig. 19) about 1.4× as long as mesosoma. Ovipositor 2.45× as long as mesotibia and strongly exserted, with the exserted part 0.35× as long as ovipositor. Third valvula 3.07× as long as mid basitarsus. Length measurements: ovipositor, 613; third valvula, 230.

***Male***. Unknown.

##### Remarks.

The new species is very close to *C. amoena* Förster 1878 in having similar colour of the body and strongly exserted ovipositor. But it can be separated from the latter by F1 with ventral margin very slightly shorter than F2 (*vs* obviously shorter, *cf.* Nikol’skaya and Yasnosh 1966, fig. 150), F2 subquadrate (*vs* about 1.8× as long as wide), clava 4.0× as long as wide (*vs* 3.5×), fore wing mostly infuscate, with a transparent cross band on the median area of disc (*vs* mostly hyaline with the area beneath marginal vein and stigmal vein infuscate).

##### Host.

Unknown.

##### Etymology.

The specific name refers to the fore wing with a wide transparent cross band on the median area of disc.

##### Distribution.

China (Heilongjiang, Shandong).

## Supplementary Material

XML Treatment for Centrodora crassiscapa

XML Treatment for Centrodora pellucida
